# Supplementary material for: Gene–Smoking Interaction Analysis for the Identification of Novel Asthma-Associated Genetic Factors
Source: Int J Mol Sci. 2023 Jul 31;24(15):12266. doi: 10.3390/ijms241512266 (PMC10419280; doi:10.3390/ijms241512266)
Supplement: Supplementary file 1 [file ijms-24-12266-s001.zip › Supplementary Figures.pdf]

Supplementary Figures

Gene–Smoking Interaction Analysis for the Identification of Novel Asthma-Associated Genetic Factors

Junho Cha [1] and Sungkyoung Choi [1,2,*]

[1] Department of Applied Artificial Intelligence, College of Computing, Hanyang University, 55 Hanyang-daehak-ro, Sangnok-gu, Ansan 15588, Republic of Korea; chajunho822@hanyang.ac.kr

[2] Department of Mathematical Data Science, College of Science and Convergence Technology, Hanyang University, 55 Hanyang-daehak-ro, Sangnok-gu, Ansan 15588, Republic of Korea

* Correspondence: day0413@hanyang.ac.kr; Tel.: +82-31-400-5465

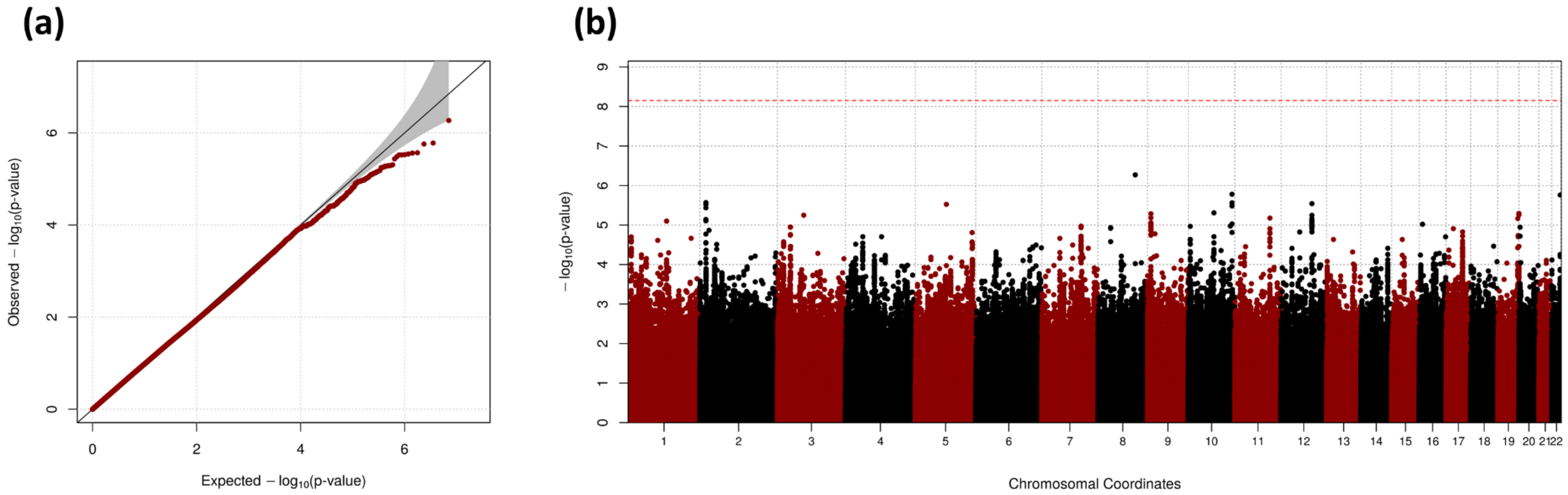

Figure S1. Quantile-quantile (Q-Q) and Manhattan plots for GWAS analysis. (**a**) Q-Q plot showing expected vs. observed $-\log_{10}(p\text{-values})$. The expected line is shown in black, and 95% confidence interval (CI) bands are shown in gray. (**b**) Manhattan plot of the *p*-values in the gene-environmental interaction analysis for asthma disease. The horizontal red line denotes the threshold for 0.05 genome-wide significance level by a Bonferroni correction of 7.08×10^{-9}.

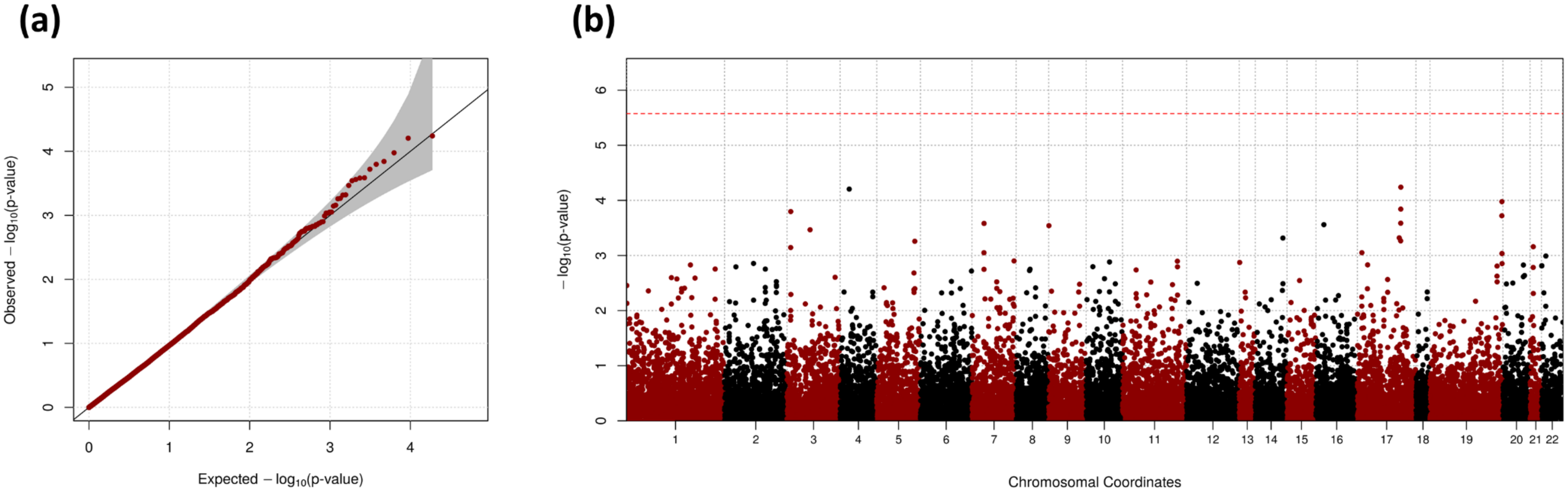

Figure S2. Quantile-quantile (Q-Q) and Manhattan plots for gene analysis. (**a**) Q-Q plot showing expected vs. observed $-\log_{10}$(*p*-values). The expected line is shown in black, and 95% confidence interval (CI) bands are shown in gray. (**b**) Manhattan plot of the *p*-values in the gene-environmental interaction analysis for asthma disease. The horizontal red line denotes the threshold for 0.05 genome-wide significance level by a Bonferroni correction of 2.66×10^{-6}.
